# Supplementary material for: Modeling glioblastoma heterogeneity as a dynamic network of cell states
Source: Mol Syst Biol. 2021 Sep 16;17(9):e10105. doi: 10.15252/msb.202010105 (PMC8444284; doi:10.15252/msb.202010105)
Supplement: Supplementary file 6 — Source Data for Figure 5 [file MSB-17-e10105-s004.zip › Figure5A_sourcedata/GSEA_3071/nosigdb_stateV.GseaPreranked.1622795159954/FELDMAN_2020_G1.html]

Details for gene set FELDMAN\_2020\_G1[GSEA]

|  || Dataset | state43071 |
| Phenotype | NoPhenotypeAvailable |
| Upregulated in class | na\_pos |
| GeneSet | FELDMAN\_2020\_G1 |
| Enrichment Score (ES) | 0.6895835 |
| Normalized Enrichment Score (NES) | 4.014703 |
| Nominal p-value | 0.0 |
| FDR q-value | 0.0 |
| FWER p-Value | 0.0 |
Table: GSEA Results Summary

  

Fig 1: Enrichment plot: FELDMAN\_2020\_G1      
 Profile of the Running ES Score & Positions of GeneSet Members on the Rank Ordered List

  

| PROBE | GENE SYMBOL | GENE\_TITLE | RANK IN GENE LIST | RANK METRIC SCORE | RUNNING ES | CORE ENRICHMENT || 1 | HIST1H1B |  |  | 0 | 2.500 | 0.0169 | Yes |
| 2 | HIST1H4C |  |  | 1 | 2.442 | 0.0333 | Yes |
| 3 | ATAD2 |  |  | 2 | 2.214 | 0.0482 | Yes |
| 4 | ESCO2 |  |  | 3 | 2.193 | 0.0630 | Yes |
| 5 | HMGB2 |  |  | 5 | 2.011 | 0.0764 | Yes |
| 6 | NUSAP1 |  |  | 6 | 2.001 | 0.0899 | Yes |
| 7 | HIST1H1C |  |  | 7 | 1.976 | 0.1032 | Yes |
| 8 | PCNA |  |  | 10 | 1.908 | 0.1157 | Yes |
| 9 | TOP2A |  |  | 14 | 1.842 | 0.1275 | Yes |
| 10 | HIST1H1E |  |  | 15 | 1.830 | 0.1398 | Yes |
| 11 | DHFR |  |  | 18 | 1.795 | 0.1515 | Yes |
| 12 | NCAPG |  |  | 21 | 1.760 | 0.1630 | Yes |
| 13 | H2AFX |  |  | 26 | 1.725 | 0.1739 | Yes |
| 14 | CENPF |  |  | 28 | 1.705 | 0.1852 | Yes |
| 15 | TYMS |  |  | 30 | 1.683 | 0.1963 | Yes |
| 16 | RRM2 |  |  | 34 | 1.660 | 0.2069 | Yes |
| 17 | CENPU |  |  | 36 | 1.645 | 0.2178 | Yes |
| 18 | MKI67 |  |  | 45 | 1.598 | 0.2270 | Yes |
| 19 | PBK |  |  | 54 | 1.536 | 0.2358 | Yes |
| 20 | GINS2 |  |  | 57 | 1.528 | 0.2457 | Yes |
| 21 | GTSE1 |  |  | 66 | 1.491 | 0.2542 | Yes |
| 22 | CDK1 |  |  | 69 | 1.488 | 0.2639 | Yes |
| 23 | PTN |  |  | 70 | 1.482 | 0.2738 | Yes |
| 24 | SMC2 |  |  | 73 | 1.466 | 0.2833 | Yes |
| 25 | SPC25 |  |  | 76 | 1.442 | 0.2927 | Yes |
| 26 | IGFBP3 |  |  | 78 | 1.437 | 0.3022 | Yes |
| 27 | UBE2T |  |  | 79 | 1.432 | 0.3118 | Yes |
| 28 | FBXO5 |  |  | 82 | 1.422 | 0.3210 | Yes |
| 29 | CLSPN |  |  | 84 | 1.417 | 0.3304 | Yes |
| 30 | SCRG1 |  |  | 87 | 1.404 | 0.3394 | Yes |
| 31 | MCM7 |  |  | 88 | 1.400 | 0.3489 | Yes |
| 32 | DEK |  |  | 94 | 1.384 | 0.3572 | Yes |
| 33 | CDCA2 |  |  | 96 | 1.382 | 0.3664 | Yes |
| 34 | RRM1 |  |  | 99 | 1.364 | 0.3752 | Yes |
| 35 | CENPM |  |  | 114 | 1.337 | 0.3815 | Yes |
| 36 | SMC4 |  |  | 127 | 1.320 | 0.3880 | Yes |
| 37 | FEN1 |  |  | 139 | 1.296 | 0.3946 | Yes |
| 38 | GMNN |  |  | 146 | 1.280 | 0.4021 | Yes |
| 39 | TMPO |  |  | 161 | 1.243 | 0.4077 | Yes |
| 40 | CENPK |  |  | 162 | 1.241 | 0.4161 | Yes |
| 41 | CDKN3 |  |  | 163 | 1.239 | 0.4245 | Yes |
| 42 | LMNB1 |  |  | 165 | 1.238 | 0.4326 | Yes |
| 43 | DNMT1 |  |  | 166 | 1.237 | 0.4409 | Yes |
| 44 | KIF23 |  |  | 167 | 1.235 | 0.4493 | Yes |
| 45 | CDCA3 |  |  | 169 | 1.230 | 0.4574 | Yes |
| 46 | GGH |  |  | 182 | 1.207 | 0.4632 | Yes |
| 47 | DBF4 |  |  | 189 | 1.197 | 0.4701 | Yes |
| 48 | KIF4A |  |  | 191 | 1.193 | 0.4779 | Yes |
| 49 | NUF2 |  |  | 192 | 1.189 | 0.4859 | Yes |
| 50 | USP1 |  |  | 202 | 1.168 | 0.4921 | Yes |
| 51 | ZWINT |  |  | 204 | 1.166 | 0.4997 | Yes |
| 52 | ASPM |  |  | 207 | 1.163 | 0.5072 | Yes |
| 53 | PRC1 |  |  | 211 | 1.161 | 0.5144 | Yes |
| 54 | ARL6IP1 |  |  | 212 | 1.158 | 0.5222 | Yes |
| 55 | KIF22 |  |  | 213 | 1.157 | 0.5300 | Yes |
| 56 | UBE2C |  |  | 214 | 1.154 | 0.5378 | Yes |
| 57 | NDC80 |  |  | 240 | 1.129 | 0.5406 | Yes |
| 58 | TPX2 |  |  | 248 | 1.122 | 0.5468 | Yes |
| 59 | RAD21 |  |  | 256 | 1.109 | 0.5529 | Yes |
| 60 | CKAP2L |  |  | 285 | 1.078 | 0.5547 | Yes |
| 61 | AURKB |  |  | 297 | 1.068 | 0.5598 | Yes |
| 62 | STMN1 |  |  | 307 | 1.062 | 0.5652 | Yes |
| 63 | CCNA2 |  |  | 309 | 1.057 | 0.5721 | Yes |
| 64 | CKAP2 |  |  | 312 | 1.053 | 0.5788 | Yes |
| 65 | PSIP1 |  |  | 313 | 1.053 | 0.5859 | Yes |
| 66 | DUT |  |  | 314 | 1.050 | 0.5930 | Yes |
| 67 | TMEM106C |  |  | 334 | 1.034 | 0.5963 | Yes |
| 68 | S100B |  |  | 361 | 0.996 | 0.5980 | Yes |
| 69 | DNAJC9 |  |  | 373 | 0.979 | 0.6024 | Yes |
| 70 | PHF19 |  |  | 378 | 0.971 | 0.6082 | Yes |
| 71 | RPA3 |  |  | 395 | 0.953 | 0.6115 | Yes |
| 72 | NUDT1 |  |  | 417 | 0.936 | 0.6138 | Yes |
| 73 | DLGAP5 |  |  | 418 | 0.936 | 0.6201 | Yes |
| 74 | MAD2L1 |  |  | 421 | 0.932 | 0.6260 | Yes |
| 75 | KPNA2 |  |  | 435 | 0.919 | 0.6296 | Yes |
| 76 | CENPE |  |  | 437 | 0.918 | 0.6356 | Yes |
| 77 | VRK1 |  |  | 445 | 0.911 | 0.6404 | Yes |
| 78 | CENPH |  |  | 448 | 0.908 | 0.6461 | Yes |
| 79 | ORC6 |  |  | 449 | 0.908 | 0.6523 | Yes |
| 80 | KNSTRN |  |  | 457 | 0.902 | 0.6570 | Yes |
| 81 | HMGN2 |  |  | 466 | 0.900 | 0.6615 | Yes |
| 82 | LSM3 |  |  | 468 | 0.892 | 0.6673 | Yes |
| 83 | UBE2S |  |  | 514 | 0.854 | 0.6644 | Yes |
| 84 | PLK1 |  |  | 526 | 0.850 | 0.6679 | Yes |
| 85 | BIRC5 |  |  | 541 | 0.842 | 0.6709 | Yes |
| 86 | CMC2 |  |  | 580 | 0.812 | 0.6690 | Yes |
| 87 | HMGB1 |  |  | 583 | 0.810 | 0.6741 | Yes |
| 88 | TK1 |  |  | 591 | 0.806 | 0.6782 | Yes |
| 89 | CENPA |  |  | 627 | 0.786 | 0.6767 | Yes |
| 90 | SIVA1 |  |  | 646 | 0.774 | 0.6784 | Yes |
| 91 | SCG2 |  |  | 682 | 0.756 | 0.6767 | Yes |
| 92 | ANP32E |  |  | 722 | 0.739 | 0.6741 | Yes |
| 93 | HAT1 |  |  | 741 | 0.727 | 0.6755 | Yes |
| 94 | AURKA |  |  | 761 | 0.718 | 0.6767 | Yes |
| 95 | DTYMK |  |  | 800 | 0.700 | 0.6741 | Yes |
| 96 | H2AFZ |  |  | 851 | 0.677 | 0.6689 | Yes |
| 97 | RNASEH2A |  |  | 859 | 0.674 | 0.6721 | Yes |
| 98 | MZT1 |  |  | 917 | 0.652 | 0.6655 | Yes |
| 99 | SRSF7 |  |  | 921 | 0.650 | 0.6693 | Yes |
| 100 | H2AFV |  |  | 933 | 0.647 | 0.6715 | Yes |
| 101 | KIF20B |  |  | 934 | 0.647 | 0.6759 | Yes |
| 102 | PTTG1 |  |  | 936 | 0.647 | 0.6800 | Yes |
| 103 | CENPN |  |  | 953 | 0.638 | 0.6812 | Yes |
| 104 | CKS2 |  |  | 966 | 0.634 | 0.6832 | Yes |
| 105 | PRCP |  |  | 983 | 0.626 | 0.6843 | Yes |
| 106 | RANBP1 |  |  | 999 | 0.620 | 0.6855 | Yes |
| 107 | TUBA1B |  |  | 1012 | 0.617 | 0.6874 | Yes |
| 108 | MIS18A |  |  | 1023 | 0.614 | 0.6896 | Yes |
| 109 | MAP3K7CL |  |  | 1055 | 0.606 | 0.6877 | No |
| 110 | HMGB3 |  |  | 1154 | 0.577 | 0.6726 | No |
| 111 | NUCKS1 |  |  | 1178 | 0.570 | 0.6719 | No |
| 112 | DEPDC1 |  |  | 1179 | 0.569 | 0.6758 | No |
| 113 | ANP32B |  |  | 1187 | 0.566 | 0.6782 | No |
| 114 | EXOSC8 |  |  | 1230 | 0.553 | 0.6738 | No |
| 115 | CCNB2 |  |  | 1373 | 0.513 | 0.6497 | No |
| 116 | DLG1 |  |  | 1491 | 0.482 | 0.6303 | No |
| 117 | CKS1B |  |  | 1502 | 0.479 | 0.6316 | No |
| 118 | CEP55 |  |  | 1508 | 0.479 | 0.6339 | No |
| 119 | TUBB4B |  |  | 1659 | 0.444 | 0.6078 | No |
| 120 | CENPW |  |  | 1959 | 0.395 | 0.5525 | No |
| 121 | LSM5 |  |  | 1982 | 0.391 | 0.5509 | No |
| 122 | MALAT1 |  |  | 1988 | 0.391 | 0.5525 | No |
| 123 | ANLN |  |  | 2125 | 0.371 | 0.5287 | No |
| 124 | HNRNPDL |  |  | 2203 | 0.362 | 0.5162 | No |
| 125 | CCNB1 |  |  | 2480 | 0.325 | 0.4649 | No |
| 126 | HNRNPA2B1 |  |  | 2523 | 0.320 | 0.4589 | No |
| 127 | BUB3 |  |  | 2806 | 0.288 | 0.4062 | No |
| 128 | CMTM7 |  |  | 4056 | -0.400 | 0.1668 | No |
| 129 | CYR61 |  |  | 4384 | -0.497 | 0.1067 | No |
| 130 | PDLIM7 |  |  | 4867 | -0.773 | 0.0185 | No |
| 131 | C12orf75 |  |  | 4940 | -0.858 | 0.0103 | No |
| 132 | IGFBP5 |  |  | 4978 | -0.897 | 0.0092 | No |
| 133 | CTGF |  |  | 5070 | -1.073 | -0.0012 | No |
| 134 | GJB2 |  |  | 5174 | -1.433 | -0.0115 | No |
| 135 | TPST2 |  |  | 5182 | -1.472 | -0.0030 | No |
| 136 | MIAT |  |  | 5225 | -1.704 | 0.0004 | No |
| 137 | AHNAK2 |  |  | 5243 | -1.927 | 0.0101 | No |
Table: GSEA details [plain text format]

  

Fig 2: FELDMAN\_2020\_G1: Random ES distribution      
 Gene set null distribution of ES for **FELDMAN\_2020\_G1**
